# Household

**Published:** 1890-06

**Authors:** 


### HOUSEHOLD.

### THE CABBAGE.

The Greeks held the cabbage in such high esteem that they deduced its origin from the father of the gods. The Saxons named February "Sprout Kale," in honor of this vegetable, so that it is appropriate to make it our February vegetable.

Chemically, according to Schrader, cabbage contains starch, vegetable albumen, resin, gum extract and some salts. Sir Humphrey Davy estimates that its nutritive matter is about 73 in one thousand parts, while Boussingault calculates that 1446 parts of white cabbage are equivalent in nutritive powers to 191 of wheat. It must be well cooked, if cooked at all, or it is less digestible than when eaten as a slaw or salade.

Phillips says that "the ancient Romans banished physicians from their territory and preserved their health 600 years by the use of cabbage." It is frequently used in long voyages to prevent scurvy and would be more effective for this purpose if cooked and eaten without pork.

How to Make and Serve Hash.—Here is a nice way to make hash of cold roast beef or steak. Use twice as much potatoes as you do of meat; chop it fine and season it highly. Place a porcelain kettle on the stove, put in it half a cup of butter, and let it get hot before putting in the hash. Add half a cup of milk and a little hot water to every quart of hash. Let it cook slowly, stirring frequently. Another nice hash is made by mixing two cups each of chopped potatoes, corned beef and toasted bread. Put half a cup of butter in a frying-pan, turn in the hash, and spread it evenly over the pan; moisten it with hot water and let it stand until it begins to be brown, then place it on a hot platter, season to taste with salt and pepper and serve immediately. If you want the dish extra nice, place eggs that have been broken into boiling water upon it just before serving it.

Fruit Cake.—Five eggs, half a cup of milk, five cups of flour, flavoring to taste, three cups of sugar, one cup of butter, ½ lb. of raisins, ½ lb. of currants. Work the butter and sugar to a cream, add the eggs well beaten, the flavoring, and sift the flour, stirring thoroughly; flour the raisins and currants, and stir in last. Bake slowly in a moderate oven for two hours.

Soups.—In making any kinds of soup, take care that your pots and pans are scrupulously clean; that all vegetables are fresh, good, and properly prepared; that your stocks are sweet and free from fat; and that you thoroughly cook and freely skim the soup during its preparation. Soft water will be found best for all soups, except those containing fresh green vegetables whose color has to be preserved. All soups will be more nourishing and tasty when made from stock instead of water, but the one may always be substituted for the other. A small quantity of celery seed tied in a piece of muslin may be used instead of celery. Soups, as a rule, are even better on the second than on the first day of cooking. They should be allowed to cool in an earthen vessel, have the scum removed, and be re-heated in a clean saucepan. Cream should never be added to any soup until just before serving, and after it has ceased to boil.

Silver Cake.—One cupful of sugar, half a cupful of butter, the whites of three eggs, half a cupful of corn starch dissolved in nearly half a cupful of milk, one and a fourth cupful of flour, half a teaspoonful of cream of tartar, one-fourth of a teaspoonful of soda, and vanilla or almond flavor. Beat the butter to a cream, and gradually beat in the sugar. Add the flavor. Mix the flour, cream of tartar and soda together, and sift. Beat the whites to a stiff froth. Add the cornstarch and milk to the beaten sugar and butter; then add the whites of the eggs and the flour. Mix quickly and thoroughly. Have the batter in sheets, and about two inches deep. Bake in moderate oven for about half an hour. A chocolate frosting is nice with this cake.

Boiling Meat.—Meat for boiling should be put into boiling water; the boiling water hardens the outside of the meat, and so keeps in the nourishing juices. After the meat has been put in, draw the pot to the side of the fire and let it simmer three or four hours, according to the size of the joint; long, gentle simmering makes the meat tender. If you were to boil it fast you would make it stringy and tasteless. It is better to buy rather coarse meat for boiling, because it is less expensive than the superior joints, and it can be made very tender by long, gentle simmering. Meat wastes less in boiling than in roasting, and you can use the water in which it has boiled for soup; so it is a decidedly economical manner of cooking.

Boiled Eggs.—It is the common way to boil eggs only about five minutes, and call them hard. They are then very "hard" of digestion. Boil ten minutes and they are still hard and soggy. Boil them twenty minutes and they become light and mealy, and may be easily mashed and seasoned. To boil eggs so that they shall be "soft," drop the whole eggs carefully into boiling water, and boil steadily three and a half minutes by the watch. This is a common method; though the white is hardened, the yolk is scarcely cooked at all. Another method is to lay the eggs in a warm basin or saucepan, and cover with boiling water. Let them remain without boiling, but where the water will keep hot for ten minutes. Both yolk and white will be cooked soft.

### AN ELEPHANT'S SELF-DENIAL.

While in England, Captain Marryat, the novelist, was intensely interested in the devotion and self-denial of a huge elephant. The beast was defending himself from swarms of mosquitoes, using a large branch to keep them from the crannies and cracks of his thick hide. His persecutors were still annoying him greatly, as was evident from his motions, when his keeper appeared with a little child. This he laid down before the animal saying: "Watch it!" and walked away. The elephant immediately broke off a small whisk from the large bough, and, instead of fanning himself, directed his attention to driving away every mosquito from the infant. He continued this until the keeper returned two hours after, thus setting, though a brute, an example of devotion which few men would have imitated.

